# One‐Pot Four‐Component Synthesis of Novel Amino‐Tetrahydro‐Chromene Derivatives Anchored with Dual Triazole Moieties

**DOI:** 10.1002/open.202500247

**Published:** 2025-07-23

**Authors:** Saeede Azhari, Mohammad Majid Mojtahedi, M. Saeed Abaee

**Affiliations:** ^1^ Department of Organic Chemistry and Natural Products Chemistry and Chemical Engineering Research Center of Iran Tehran 14968-13151 Iran

**Keywords:** 4H‐chromenes, click chemistry, four‐component reaction, heterocyclic chemistry, ultrasonic irradiation

## Abstract

A double aldol condensation‐Michael addition‐cyclization‐double click reaction sequence is conducted in one pot for the synthesis of a novel series of tetrahydro‐chromene derivatives anchored with dual triazole rings. The process is optimized primarily step by step under ultrasonic irradiation in a basic aqueous *t*‐BuOH medium. The steps are then successfully combined into a four‐component one‐pot reaction using the optimum conditions, where the whole operation took less than 2 h to complete. As a result, the new products are precipitated in the reaction vessel and obtained directly by simple filtration–crystallization without undergoing any other costly separation or chromatographic operations. The structures of the intermediates and the products are characterized using various spectroscopic methods.

## Graphical abstract

A series of tetrahydrochromene derivatives bearing triazole rings is synthesized via a one‐pot reaction. The process is optimized by ultrasonication of a basic aqueous *tert*‐butanol mixture. Subsequently, the optimized steps are successfully integrated into a four‐component, one‐pot protocol. The whole process was completed in less than 2 h. Products precipitated directly in the reaction vessel and are easily isolated by simple filtration and crystallization.

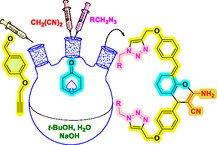

## 1 Introduction

The globally enforced sustainability mandates in chemical production^[^ ^1^ ^]^ require modern synthesis to get involved severely with efficiency and green criteria.^[^ ^2^ ^]^ In this context, a breakthrough solution is multicomponent reaction (MCR) approaches,^[^ ^3^, ^4^ ^]^ emerged with significant advantages such as improved energy efficiency, atom economy, and minimized waste production,^[^ ^5^ ^]^ avoiding separation and characterization of the intermediate products and thus retrenching the resources and labor efforts.^[^ ^6^ ^]^ Significant recent instances include Michael–Michael–aldol,^[^ ^7^, ^8^ ^]^ aldol condensation‐Michael addition‐Suzuki coupling,^[^ ^9^ ^]^ Knoevenagel condensation‐1,3‐dipolar cycloaddition,^[^ ^10^ ^]^ aldol‐Knoevenagel‐Diels‐Alder,^[^ ^11^ ^]^ Michael‐Michael‐aldol‐β‐lactonization,^[^ ^12^ ^]^ and many other related domino reactions.^[^ ^13^, ^14^, ^15^ ^]^

**Figure 1:**
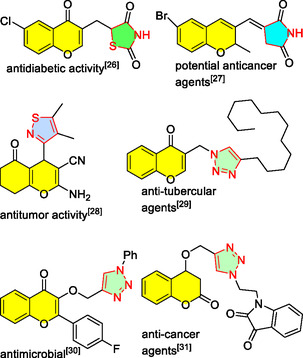
Various roles of DBU in organic synthesis.^[^ ^37^, ^38^, ^39^, ^40^, ^41^, ^42^ ^]^

An important group of oxygen‐containing compounds are the chromene heterocycles,^[^ ^16^ ^]^ which are highlighted in many cases with their intriguing biological^[^ ^17^ ^]^ and pharmaceutical activities.^[^ ^18^ ^]^ In addition, they constitute naturally occurring compounds in the form of tocopherols,^[^ ^19^ ^]^ flavones,^[^ ^20^ ^]^ alkaloids,^[^ ^21^ ^]^ and anthocyanins.^[^ ^22^ ^]^ Thus, chromene derivatives are always of interest for possibly exhibiting useful biochemical^[^ ^23^ ^]^ or medicinal^[^ ^24^ ^]^ properties, and the synthesis of their new derivatives is always in demand. A genuine approach in this context is combining the 4H‐chromene moiety with other biologically active heterocyclic fragments into molecular scaffolds with improved potentials higher than those of the starting individual components.^[^ ^25^ ^]^ Properly designed combinations would then lead to new molecular structures that synergistically possess enhanced desired properties. Some important recent examples in which the 4H‐chromene core is coupled with various azole heterocycles are highlighted in **Figure** **1**.

**Scheme 1:**
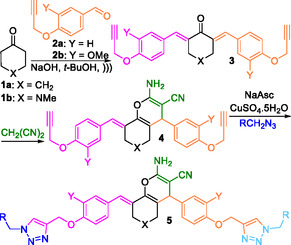
General process pathway for one‐pot synthesis of **5**.

Appropriate candidates to hybrid with 4H‐chromene for the synthesis of new useful structures include azoles with their five‐membered nitrogen‐containing rings (as exemplified in Figure 1), among which triazoles have been the most frequently practiced moieties in synthetic chemistry in recent decades^[^ ^26^ ^]^ and are usually obtained through Click chemistry.^[^ ^27^ ^]^ The skeleton offers the privilege of nitrogen heteroatoms in the ring, which are very important from structural and biological points of view.^[^ ^28^ ^]^ These are the reasons for a considerable extension of the chemistry and biology of triazole‐containing structures.^[^ ^29^ ^]^ Due to these features and in the framework of our program to study the heterocyclic chemistry^[^ ^30^, ^31^ ^]^ and MCRs,^[^ ^32^, ^33^ ^]^ we were persuaded to develop new 4H‐chromene‐triazole structural hybrids based on appropriately derivatized bisarylmethyledenes of the 6‐membered cyclic ketones. The idea is depicted in **Scheme** **1** for one‐pot combination of ketones **1** with malononitrile and different azides leading to direct synthesis of derivatives of **5**.

## 2 Results and Discussion

First, we optimized the model reaction by examining various conditions for the combination of **1a** with 4‐(prop‐2‐yn‐1‐yloxy)benzaldehyde **2a** (*Y* = *H*) (**Table** **1**). Under NaOH_aq_/t‐BuOH conditions, **3a** was formed in 94% after 20 min of ultrasonic irradiation (entry 1). In the absence of either the irradiation (entry 2) or the base (entry 3), the yield diminished considerably. Further optimization of the reaction was carried out by variation in the amounts and the type of the base. The changes in the amounts of the base (entries 4–6) showed 45 mol% as the optimum molar ratio. The use of other inorganic (entries 7–8) or amine bases (entries 9–11) did not improve the outcome. This was also the case when t‐BuOH was replaced with other solvents (entries 12–16). These optimum conditions [NaOH_aq_ (45 mol%)/t‐BuOH] were then used for the next step to combine **3a** with malononitrile, giving 95% of **4a** within 5 min of irradiation (entry 17). By the addition of CuSO_4_ and sodium ascorbate (NaAsc), the conditions were shown to be amenable to efficiently convert the mixture of **4a** + PhCH_2_N_3_ to **5a** (entry 18). Finally, we completed the optimization experiments by conducting all three steps in the same pot. Thus, when all steps were subsequently run one after another, **5a** was obtained directly by precipitation in the reaction vessel in high yields (entry 19).

**Table 1:** Optimization of the synthesis of 3a–5a.

Entry	Reactants	Conditions	Time (min)	Product	Yield [%]a)
1	**1** + **2a**	NaOH_aq_ (45 mol%)/t‐BuOH	20	**3a**	94
2	**1** + **2a**	NaOH_aq_ (45 mol%)/t‐BuOH	20	**3a**	10
3	**1 **+ **2a**	t‐BuOH	20	**3a**	0
4	**1** + **2a**	NaOH_aq_ (60 mol%)/t‐BuOH	20	**3a**	84
5	**1** + **2a**	NaOH_aq_ (30 mol%)/t‐BuOH	20	**3a**	70
6	**1** + **2a**	NaOH_aq_ (20 mol%)/t‐BuOH	20	**3a**	56
7	**1** + **2a**	KOH_aq_ (45 mol%)/t‐BuOH	20	**3a**	79
8	**1** + **2a**	K_2_CO_3aq_ (45 mol%)/t‐BuOH	20	**3a**	45
9	**1** + **2a**	Et_3_N/t‐BuOH	20	**3a**	33
10	**1** + **2a**	pyrrolidine/t‐BuOH	20	**3a**	24
11	**1 **+ **2a**	DABCO/t‐BuOH	20	**3a**	48
12	**1 **+ **2a**	NaOH_aq_ (45 mol%)/ EtOH	20	**3a**	70
13	**1** + **2a**	NaOH_aq_ (45 mol%)/ MeOH	20	**3a**	61
14	**1** + **2a**	NaOH_aq_ (45 mol%)/ DMF	20	**3a**	38
15	**1** + **2a**	NaOH_aq_ (45 mol%)/ MeCN	20	**3a**	21
16	**1** + **2a**	NaOH_aq_ (45 mol%)/ CH_2_Cl_2_	20	**3a**	12
17	**3a** + CH_2_(CN)_2_	NaOH_aq_ (45 mol%)/t‐BuOH	5	**4a**	95
18	**4a** + PhCH_2_N_3_	CuSO_4_.5H_2_O (8 mol%), NaAsc/t‐BuOH_aq_	40	**5a**	91
19	**1** + **2a** + CH_2_(CN)_2_ + PhCH_2_N_3_	1) NaOH_aq_ (45 mol%)/t‐BuOH 2) CH_2_(CN)_2_ 3) CuSO_4_.5H_2_O (8 mol%), NaAsc/t‐BuOH_aq_ PhCH_2_N_3_	65	**5a**	87

a) Isolated yields.

Next, we applied the optimum conditions to evaluate the synthetic scope of the method by using other derivatives of the reactants (**Table** **2**). Therefore, in addition to the reaction with **2a** (*Y* = H), malononitrile, and PhCH_2_N_3_ leading to the synthesis of **5a** (entry 1), cyclohexanone **1a** was reacted with **2a and b,** and other derivatives of the azide reagent as well to obtain **5b–d** in high yields and within short time periods (entries 2–4). Similarly, the reaction of the heterocyclic analog of the starting ketone (**1b**) with the other reactants gave **5e–h** efficiently (entries 5–8). In all cases, products were obtained directly by precipitation and crystallization from EtOH, and no chromatographic separations were needed.

**Table 2:** Synthesis of various derivatives of 5.

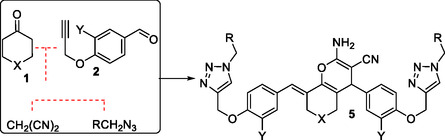						
entry	*X*	*Y*	*R*	Time [min]	Product	Yield [%]a)
1	CH_2_	H	C_6_H_5_	65	**5a**	87
2	CH_2_	H	4‐MeC_6_H_4_	70	**5b**	90
3	CH_2_	H	cyclopropyl	90	**5c**	87
4	CH_2_	OMe	C_6_H_5_	70	**5d**	89
5	NMe	H	C_6_H_5_	75	**5e**	90
6	NMe	H	4‐MeC_6_H_4_	50	**5f**	88
7	NMe	OMe	4‐BrC_6_H_4_	55	**5g**	91
8	NMe	OMe	4‐MeC_6_H_4_	65	**5h**	85

a) Isolated yields.

**Figure 2:**
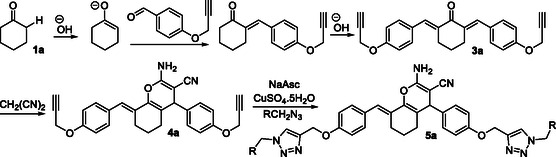
The proposed mechanism of the process.

Based on the results, a mechanism would be proposed for the reaction, as presented in **Figure** **2**, starting from **1a**. In the presence of the base, **1a** is converted to the corresponding enolate, which would undergo two sequential aldol condensation reactions with the propargylic aldehyde to afford **3a**. Next, the nucleophilic attack of malononitrile on **3a,** followed by cyclization of the corresponding intermediate, would produce the required pyran ring. In the last step, double cyclization of **4a** with two equivalents of the azide would give the final product **5a**.

## 3 Conclusion

In summary, we carried out a four‐component reaction for the synthesis of novel target products containing either the 4H‐chromene or the 4H‐pyranopyridine central rings. The process takes place through an aldol condensation‐Michael addition‐cyclization‐click sequence of reactions via a one‐pot route, and the products are obtained directly as precipitates in the reaction vessel without performing any costly chromatographic operations. The study of the biological properties of the products and the application of the method to other heterocyclic systems would be investigated in due course.

## 4 Experimental Section

4.1

4.1.1

##### General

Chemicals and starting materials were purchased from commercial sources. The starting aldehydes^[^ ^34^, ^35^ ^]^ and the azides^[^ ^36^ ^]^ were prepared using available methods. TLC experiments were carried out on precoated silica gel plates using hexanes/EtOAc (6:1) as the eluent. Melting points are uncorrected. FT‐IR spectra were recorded using KBr disks on a Bruker Vector‐22 infrared spectrometer. NMR spectra were obtained on a FT‐NMR Bruker UltraShield (500 MHz for ^1^H and 125MHz for ^13^C) as CDCl_3_ solutions using TMS as the internal standard reference. Elemental analyses were performed using a Thermo Finnigan Flash EA 1112 instrument. MS spectra were obtained on a Fisons 8000 Trio instrument at an ionization potential of 70 eV. The new products were identified based on their full spectroscopic analyses.

##### Typical One‐Pot Synthesis of **5a**

A mixture of cyclohexanone **1a** (98 mg, 1.0 mmol), **2a** (320 mg, 2.0 mmol), and NaOH_aq_ (60 μl, 30 mol%) in *t*‐BuOH/H_2_O (1:1, 2.0 mL) was sonicated in a 25 mL flask for 20 min until TLC showed the formation of the intermediate **3a**. At this point, malononitrile (100 mg, 1.5 mmol) was added to the mixture, and sonication was continued for another 5 min until intermediate **3a** was completely consumed and the formation of **4a** was eventually detected. At this point, the benzyl azide (293 mg, 2.2 mmol), CuSO_4_.5H_2_O (40 mg, 0.16 mmol), and sodium ascorbate (79 mg, 0.40 mmol) were added to the mixture, and sonication was continued for another 40 min until TLC indicated the completion of the reaction. The product that precipitated in the mixture was filtered and recrystallized from EtOH to obtain the final **5a** (621 mg, 87%) as a white solid.

##### Spectral Data of the New Products: (E)‐2‐Amino‐8‐(4‐(prop‐2‐yn‐1‐yloxy)Benzylidene)‐4‐(4‐(prop‐2‐yn‐1‐yloxy)Phenyl)‐5,6,7,8‐tetrahydro‐4H‐chromene‐3‐carbonitrile (**4a**)

White powder; m.p: 269–271 °C; ^1^H NMR (500 MHz, DMSO‐d_6_) *δ* 7.26 (d, *J* *=* 8.5 Hz, 2H), 7.11 (d, *J* *=* 8.5 Hz, 2H), 6.98 (d, *J* = 8.5 Hz, 2H), 6.95 (d, *J* iki = 8.5 Hz, 2H), 6.89 (s, 1H), 6.72 (s, 2H), 4.79 (s, 2H), 4.76 (s, 2H), 3.90 (s, 1H), 3.57–3.55 (m, 2H), 2.63–2.61 (m, 1H), 2.48–2.47 (m, 1H), 2.04–1.99 (m, 1H), 1.80–1.76 (m, 1H), and 1.55–1.50 (m, 2H) ppm; ^13^C NMR (125 MHz, DMSO‐d_6_) *δ* 160.2, 156.6, 156.5, 142.4, 137.3, 130.8, 130.4, 129.1, 128.5, 122.0, 121.2, 115.4, 115.3, 115.2, 79.8, 79.7, 78.7, 78.6, 56.6, 55.9, 55.8, 42.9, 27.2, 27.0, and 22.3 ppm; IR (KBr, cm^−1^) 3449, 3355, 2210, 1627, and 627; MS (70 eV) *m*/*z* 448 [M^+^], 317, 256, and 129; Anal. Calcd for C_29_H_24_N_2_O_3_: C, 77.66; H, 5.39; N, 6.25; Found: C, 77.76; H, 5.49; N, 6.11.

##### (E)‐2‐Amino‐8‐(4‐((1‐benzyl‐1H‐1,2,3‐triazol‐4‐yl)methoxy)benzylidene)‐4‐(4‐((1‐benzyl‐1H‐1,2,3‐triazol‐4‐yl)methoxy)phenyl)‐5,6,7,8‐tetrahydro‐4H‐chromene‐3‐carbonitrile (**5a**)

White powder; m.p: 169–171 °C; ^1^H NMR (500 MHz, DMSO‐d_6_) *δ* 8.25 (s, 2H), 7.34–7.33 (m, 4H), 7.29–7.27 (m, 6H), 7.22 (d, *J* = 8.5 Hz, 2H), 7.08 (d, *J* = 8.5 Hz, 2H), 7.00 (d, *J* = 8.5 Hz, 2H), 6.96 (d, *J* = 8.5 Hz, 2H), 6.87 (s, 1H), 6.69 (s, 2H), 5.58 (s, 4H), 5.12 (s, 2H), 5.09 (s, 2H), 3.87 (s, 1H), 2.46–2.44 (m, 2H), 2.00–1.97 (m, 1H), 1.77–1.73 (m, 1H), and 1.47–1.46 (m, 2H) ppm; ^13^C NMR (125 MHz, DMSO‐d_6_) *δ* 160.4, 157.7, 157.5, 148.5, 143.7, 143.6, 141.2, 137.5, 137.0, 136.6, 131.0, 130.2, 129.4, 129.2, 128.8, 128.6, 128.4, 125.3, 125.2, 124.4, 122.2, 122.1, 121.3, 115.5, 115.4, 115.2, 61.8, 61.7, 56.9, 53.6, 53.5, 43.0, 27.3, 27.1, and 22.5 ppm; IR (KBr, cm^−1^) 3368, 2936, 2184, 1505, and 1245; MS (70 eV) m/z 714 [M^+^], 473, 268, 144, 91, Anal. Calcd for C_43_H_38_N_8_O_3_: C, 72.25; H, 5.36; N, 15.68; Found: C, 72.12; H, 5.46; N, 15.78.

##### (E)‐2‐Amino‐8‐(4‐((1‐(4‐methylbenzyl)‐1H‐1,2,3‐triazol‐4‐yl)methoxy)benzylidene)‐4‐(4‐((1‐(4‐methylbenzyl)‐1H‐1,2,3‐triazol‐4‐yl)methoxy)phenyl)‐5,6,7,8‐tetrahydro‐4H‐chromene‐3‐carbonitrile (**5b**)

White powder; m.p: 147–148 °C; ^1^H NMR (500 MHz, DMSO‐d_6_) *δ* 8.21 (s, 2H), 7.23–7.21 (m, 6H), 7.14–7.13 (m, 4H), 7.07–7.06 (m, 2H), 6.98–6.97 (m, 4H), 6.86 (s, 1H), 6.69 (s, 2H), 5.52 (s, 4H), 5.10 (s, 2H), 5.07 (s, 2H), 3.86 (s, 1H), 2.58–2.57 (m, 2H), 2.24 (s, 6H), 1.99–1.98 (m, 1H), 1.76–1.75 (m, 1H), and 1.48–1.47 (m, 2H) ppm; ^13^C NMR (125 MHz, DMSO‐d_6_) *δ* 160.4, 157.7, 157.5, 157.4, 143.7, 143.5, 141.2, 138.1, 137.0, 133.6, 131.0, 130.2, 129.9, 129.8, 129.7, 129.2, 128.6, 128.4, 125.1, 125.0, 122.2, 122.1, 121.3, 115.5, 115.4, 115.3, 115.2, 61.8, 61.7, 56.8, 53.3, 43.0, 27.3, 27.2, 27.1, 22.5, and 21.3 ppm; IR (KBr, cm^−1^) 3368, 2929, 2185, 1507, and 1247; MS (70 eV) *m*/*z* 742 [M^+^], 387, 201, 158, and 105; Anal. Calcd for C_45_H_42_N_8_O_3_: C, 72.76; H, 5.70; N, 15.08. Found: C, 72.91; H, 5.97; N, 15.30.

##### (E)‐2‐Amino‐8‐(4‐((1‐(cyclopropylmethyl)‐1H‐1,2,3‐triazol‐4‐yl)methoxy)benzylidene)‐4‐(4‐((1‐(cyclopropylmethyl)‐1H‐1,2,3‐triazol‐4‐yl)methoxy)phenyl)‐5,6,7,8‐tetrahydro‐4H‐chromene‐3‐carbonitrile (**5c**)

White powder; m.p: 171–173 °C; ^1^H NMR (500 MHz, DMSO‐d_6_) *δ* 8.28 (s, 1H), 8.27 (s, 1H), 7.28 (d, *J* = 8.5 Hz, 2H), 7.14 (d, *J* = 8.5 Hz, 2H), 7.08 (d, *J* = 8.5 Hz, 2H), 7.03 (d, *J* = 8.5 Hz, 2H), 6.92 (s, 1H), 6.73 (s, 2H), 5.17 (s, 2H), 5.14 (s, 2H), 4.26 (s, 2H), 4.24 (s, 2H), 3.92 (s, 1H), 2.67–2.63 (m, 1H), 2.48–2.47 (m, 1H), 2.06–2.01 (m, 1H), 1.82–1.79 (m, 1H), 1.56–1.52 (m, 2H), 1.30–1.29 (m, 2H), 0.57–0.56 (m, 2H), 0.56–0.54 (m, 2H), 0.44–0.43 (m, 2H), and 0.42–0.41 (m, 2H) ppm; ^13^C NMR (125 MHz, DMSO‐d_6_) *δ* 160.2, 157.6, 157.4, 143.1, 143.0, 141.0, 136.8, 130.9, 130.0, 129.7, 128.2, 124.6, 124.5, 122.0, 121.1, 115.3, 115.1, 115.0, 61.6, 61.5, 60.2, 56.6, 56.5, 55.3, 54.2, 42.8, 27.1, 27.0, 22.3, 19.0, 14.5, 11.8, and 4.2 ppm; IR (KBr, cm^−1^) 3365, 2914, 2184, 1505, and 1248; MS (70 eV) *m*/*z* 642 [M^+^], 305, 201, 115. Anal. Calcd for C_37_H_38_N_8_O_3_: C, 69.14; H, 5.96; N, 17.43. Found: C, 68.90; H, 6.12; N, 17.29.

##### (E)‐2‐Amino‐8‐(4‐((1‐benzyl‐1H‐1,2,3‐triazol‐4‐yl)methoxy)‐3‐methoxybenzylidene)‐4‐(4‐((1‐benzyl‐1H‐1,2,3‐triazol‐4‐yl)methoxy)‐3‐methoxyphenyl)‐5,6,7,8‐tetrahydro‐4H‐chromene‐3‐carbonitrile (**5d**)

White powder; m.p: 140–142 °C; ^1^H NMR (500 MHz, DMSO‐d_6_) *δ* 8.30 (s, 1H), 8.29 (s, 1H), 7.39–7.38 (m, 4H), 7.36–7.34 (m, 6H), 7.15 (d, *J* = 8.0 Hz, 1H), 7.12 (d, *J* = 8.0 Hz, 1H) 6.92–6.90 (m, 2H), 6.87 (d, *J* = 8.5 Hz, 1H), 6.80 (s, 1H), 6.74–6.73 (m, 3H), 5.64 (s, 4H), 5.14 (s, 2H), 5.12 (s, 2H), 3.94 (s, 1H), 3.75 (s, 3H), 3.73 (s, 3H), 2.71–2.68 (m, 1H), 2.07–2.03 (m, 1H), 1.86–1.82 (m, 1H), 1.56–1.53 (m, 2H), and 1.26–1.25 (m, 1H) ppm; ^13^C NMR (125 MHz, DMSO‐d_6_) *δ* 159.8, 149.0, 148.7, 146.5, 146.4, 143.1, 143.0, 140.6, 137.2, 136.0, 130.2, 128.7, 128.2, 128.1, 128.0, 127.9, 127.8, 124.8, 124.7, 124.3, 121.9, 121.5, 120.7, 119.5, 114.9, 114.0, 113.9, 113.7, 113.6, 113.1, 111.7, 61.9, 61.8, 56.1, 55.5, 55.4, 52.8, 42.7, 26.7, 26.6, and 22.0 ppm; IR (KBr, cm^−1^) 3323, 2936, 2192, 1513, 1255; MS (70 eV) *m*/*z* 774 [M^+^], 551, 462, 313, 144, Anal. Calcd for C_45_H_42_N_8_O_5_: C, 69.75; H, 5.46; N, 14.46. Found: C, 69.97; H, 5.70; N, 14.65.

##### (E)‐2‐Amino‐8‐(4‐((1‐benzyl‐1H‐1,2,3‐triazol‐4‐yl)methoxy)benzylidene)‐4‐(4‐((1‐benzyl‐1H‐1,2,3‐triazol‐4‐yl)methoxy)phenyl)‐6‐methyl‐5,6,7,8‐tetrahydro‐4H‐pyrano[3,2‐c]pyridine‐3‐carbonitrile (**5e**)

White powder; m.p: 134–136 °C; ^1^H NMR (500 MHz, DMSO‐d_6_) *δ* 8.31 (s, 2H), 7.40–7.39 (m, 4H), 7.36–7.34 (m, 6H), 7.22 (d, *J* = 8.5 Hz, 2H), 7.15 (d, *J* = 8.5 Hz, 2H), 7.08 (d, *J* = 8.5 Hz, 2H), 7.03 (d, *J* = 8.5 Hz, 2H), 6.88 (s, 1H), 6.81 (s, 2H), 5.64 (s, 4H), 5.18 (s, 2H), 5.15 (s, 2H), 4.02 (s, 1H), 3.48 (d, *J* = 14.0 Hz, 1H), 3.29 (d, *J* = 14.0 Hz, 1H), 3.00 (d, *J *= 14.0 Hz, 1H), 2.58 (d, *J* = 14.0 Hz, 1H), and 2.17 (s, 3 H) ppm; ^13^C NMR (125 MHz, DMSO‐d_6_) *δ* 159.7, 157.2, 157.1, 143.1, 143.0, 142.9, 139.1, 139.0, 136.0, 135.9, 135.8, 130.3, 128.8, 128.7, 128.6, 128.1, 128.0, 127.9, 126.1, 126.0, 124.7, 124.6, 120.9, 120.5, 114.8, 114.7, 112.7, 61.1, 61.0, 54.5, 54.2, 53.0, 52.9, 44.5, and 40.3 ppm; IR (KBr, cm^−1^) 3431, 2952, 2213, 1611, and 1271; MS (70 eV) *m*/*z* 729 [M^+^], 360, 216, 144; Anal. Calcd for C_43_H_39_N_9_O_3_: C, 70.76; H, 5.39; N, 17.27. Found: C, 70.88; H, 5.49; N, 17.12.

##### (E)‐2‐Amino‐6‐methyl‐8‐(4‐((1‐(4‐methylbenzyl)‐1H‐1,2,3‐triazol‐4‐yl)methoxy)benzylidene)‐4‐(4‐((1‐(4‐methylbenzyl)‐1H‐1,2,3‐triazol‐4‐yl)methoxy)phenyl)‐5,6,7,8‐tetrahydro‐4H‐pyrano[3,2‐c]pyridine‐3‐carbonitrile (**5f**)

White powder; m.p: 139–141 °C; ^1^H NMR (500 MHz, DMSO‐d_6_) *δ* 8.21 (s, 2H), 7.19–7.18 (m, 6H), 7.15–7.13 (m, 6H), 7.03–7.01 (m, 2H), 6.97–6.96 (m, 2H), 6.82 (s, 1H), 6.76 (s, 2H), 5.52 (s, 4H), 5.11 (s, 2H), 5.08 (s, 2H), 3.95 (s, 1H), 3.43–341 (m, 1H), 3.33‐.31 (m, Hz, 1H), 2.93–292 (m, 1H), 2.45–2.44 (m, 1H), 2.24 (s, 6H), and 2.10 (s, 3H) ppm; ^13^C NMR (125 MHz, DMSO‐d_6_) *δ* 160.4, 157.8, 157.7, 143.7, 143.5, 139.8, 138.1, 138.0, 136.5, 133.6, 131.0, 129.9, 129.8, 129.5, 129.4, 129.3, 129.2, 128.6, 128.5, 126.6, 125.1, 125.0, 121.6, 121.1, 115.4, 115.3, 113.2, 61.8, 61.7, 56.9, 55.2, 54.8, 53.3, 45.1, 41.0, 21.3, and 21.2 ppm; IR (KBr, cm^−1^) 3317, 2936, 2190, 1508, and 1247; MS (70 eV) *m*/*z* 757 [M^+^], 523, 368, 216, 158; Anal. Calcd for C_45_H_43_N_9_O_3_: C, 71.31; H, 5.72; N, 16.63; Found: C, 71.21; H, 5.93; N, 16.80.

##### (E)‐2‐Amino‐8‐(4‐((1‐(4‐bromobenzyl)‐1H‐1,2,3‐triazol‐4‐yl)methoxy)‐3‐methoxybenzylidene)‐4‐(4‐((1‐(4‐bromobenzyl)‐1H‐1,2,3‐triazol‐4‐yl)methoxy)‐3‐methoxyphenyl)‐6‐methyl‐5,6,7,8‐tetrahydro‐4H‐pyrano[3,2‐c]pyridine‐3‐carbonitrile (**5g**)

White powder; m.p: 163–164 °C; ^1^H NMR (500 MHz, DMSO‐d_6_) *δ* 8.46 (s, 2H), 7.57–7.56 (m, 4H), 7.53–7.51 (m, 6H), 7.35–7.29 (m, 2H), 7.05–7.04 (m, 1H), 7.03–6.99 (m, 3H), 6.92 (s, 1H), 5.81 (s, 4H), 5.32 (s, 2H), 5.29 (s, 2H), 4.19 (s, 1H), 3.93 (s, 3H), 3.90 (s, 3H), 3.71 (d, *J* = 14.0 Hz, 1H), 3.49–3.48 (m, 1H), 3.16 (d, *J *= 16.0 Hz, 1H), 2.76 (d, *J* = 16.0 Hz, 1H), and 2.35 (s, 3 H) ppm; ^13^C NMR (125 MHz, DMSO‐d_6_) *δ* 160.4, 149.7, 149.5, 147.4, 147.2, 143.7, 143.6, 139.8, 137.3, 136.6, 130.0, 129.4, 129.3, 129.2, 128.6, 128.5, 128.3, 126.8, 125.4, 125.3, 122.1, 122.0, 121.9, 121.1, 120.3, 120.2, 114.6, 114.3, 113.7, 113.1, 112.3, 62.5, 62.4, 56.8, 56.2, 56.1, 55.2, 54.8, 53.5, 45.1, and 41.3 ppm; IR (KBr, cm^−1^) 3366, 2927, 2186, 1506, and 1245; MS (70 eV) *m/z* 947 [M^+^], 523, 342, 246, and 144; Anal. Calcd for C_45_H_41_Br_2_N_9_O_5_: C, 57.03; H, 4.36; N, 13.30. Found: C, 56.80; H, 4.45; N, 13.39.

##### (E)‐2‐Amino‐8‐(3‐methoxy‐4‐((1‐(4‐methylbenzyl)‐1H‐1,2,3‐triazol‐4‐yl)methoxy)benzylidene)‐4‐(3‐methoxy‐4‐((1‐(4‐methylbenzyl)‐1H‐1,2,3‐triazol‐4‐yl)methoxy)phenyl)‐6‐methyl‐5,6,7,8‐tetrahydro‐4H‐pyrano[3,2‐c]pyridine‐3‐carbonitrile (**5h**)

White powder; m.p: 171–173 °C; ^1^H NMR (500 MHz, DMSO‐d_6_) *δ* 8.24 (s, 1H), 8.23 (s, 1H), 7.24 (d, *J* = 8.5 Hz, 2H), 7.23 (d, *J* = 8.5 Hz, 2H), 7.19–7.14 (m, 4H), 7.15 (d, *J* = 8.5 Hz, 1H), 7.11 (d, *J* = 8.5 Hz, 1H), 6.87 (s, 1H), 6.84 (s, 1H), 6.81–6.80 (m, 2H), 6.79 (s, 2H), 6.74–6.72 (m, 1H), 5.56 (s, 4H), 5.13 (s, 2H), 5.10 (s, 2H), 4.01 (s, 1H), 3.74 (s, 3H), 3.72 (s. 3H), 3.50 (d, *J* = 14.0 Hz, 1H), 3.30 (d, *J* = 14.0 Hz, 1H), 2.58 (d, *J* = 14.0 Hz, 1H), 2.28 (s, 6H), 2.42–2.23 (m, 1H), and 2.16 (s, 3H) ppm; ^13^C NMR (125 MHz, DMSO‐*d*_6_) *δ* 160.2, 149.4, 149.2, 147.2, 147.1, 143.5, 143.4, 139.6, 138.0, 137.1, 133.5, 133.4, 129.9, 129.8, 129.7, 128.5, 128.4, 126.6, 126.5, 125.2, 125.1, 121.9, 121.8, 121.0, 120.1, 119.3, 114.3, 114.0, 113.4, 113.0, 112.0, 62.2, 62.1, 56.6, 56.0, 55.9, 55.0, 54.7, 53.1, 45.0, 41.2, 21.2, 21.0 ppm; IR (KBr, cm^−1^) 3323, 2935, 2189, 1511, and 1253; MS (70 eV) *m*/*z* 817 [M^+^], 523, 368, 236, and 158; Anal. Calcd for C_47_H_47_N_9_O_5_: C, 69.02; H, 5.79; N, 15.41. Found: C, 69.21; H, 5.94; N, 15.17.

## Conflict of Interest

The authors declare no conflict of interest.

## Supporting information

Supplementary Material

## Data Availability

The data that support the findings of this study are available from the corresponding author upon reasonable request.
