# Supplementary material for: One‐Pot Four‐Component Synthesis of Novel Amino‐Tetrahydro‐Chromene Derivatives Anchored with Dual Triazole Moieties
Source: ChemistryOpen. 2025 Jul 23;14(11):e202500247. doi: 10.1002/open.202500247 (PMC12598799; doi:10.1002/open.202500247)
Supplement: Supplementary file 1 — Supplementary Material [file OPEN-14-e202500247-s001.pdf]

One-Pot Four-Component Synthesis of Novel Amino-tetrahydro-chromene Derivatives Anchored with Dual Triazole Moieties

Saeede Azhari, Mohammad M. Mojtahedi, M. Saeed Abaee

Department of Organic Chemistry and Natural Products, Chemistry and Chemical Engineering Research Center of Iran

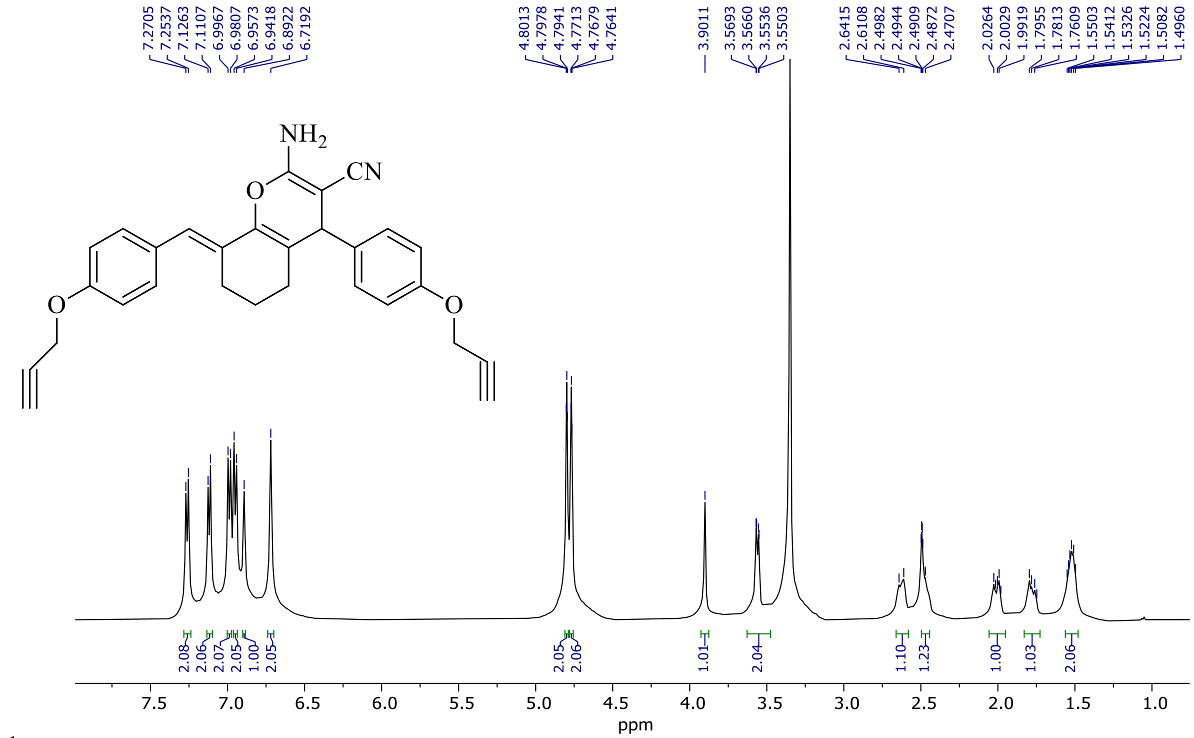

^{1}H NMR (500 MHz, DMSO-d_6) spectrum of (*E*)-2-amino-8-(4-(prop-2-yn-1-yloxy)benzylidene)-4-(4-(prop-2-yn-1-yloxy)phenyl)-5,6,7,8-tetrahydro-4*H*-chromene-3-carbonitrile (**4a**)

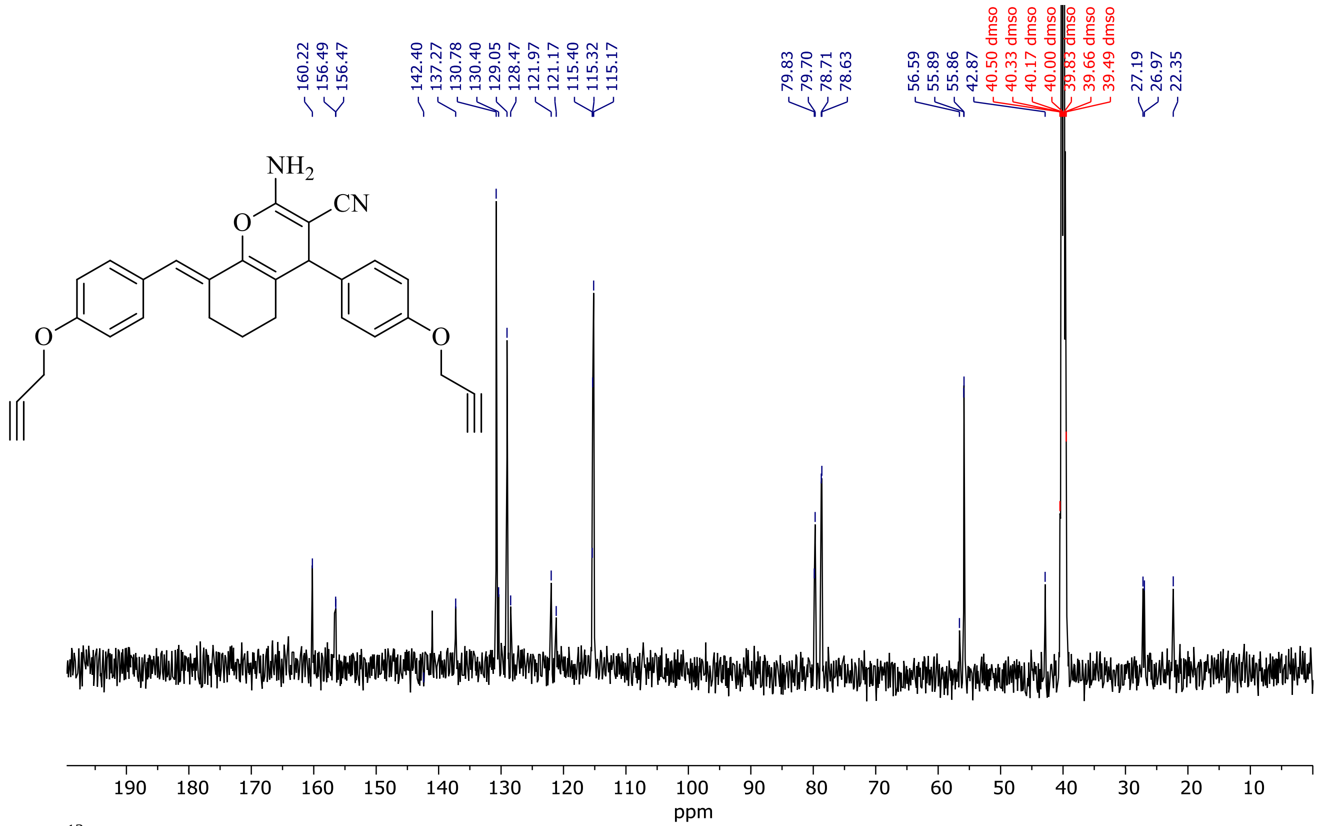

^{13}C NMR (125 MHz, DMSO-d_6) spectrum of (*E*)-2-amino-8-(4-(prop-2-yn-1-yloxy)benzylidene)-4-(4-(prop-2-yn-1-yloxy)phenyl)-5,6,7,8-tetrahydro-4*H*-chromene-3-carbonitrile (**4a**)

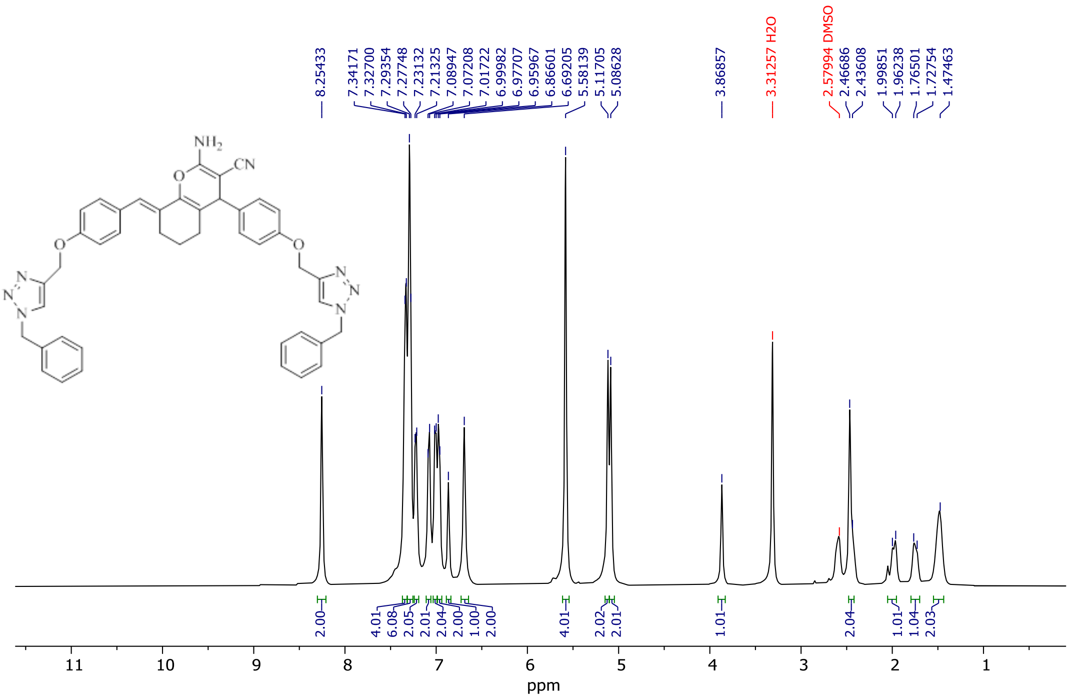

^{1}H NMR (500 MHz, DMSO-d_6) spectrum of compound (*E*)-2-amino-8-(4-((1-benzyl-1*H*-1,2,3-triazol-4-yl)methoxy)benzylidene)-4-(4-((1-benzyl-1*H*-1,2,3-triazol-4-yl)methoxy)phenyl)-5,6,7,8-tetrahydro-4*H*-chromene-3-carbonitrile (**5a**)

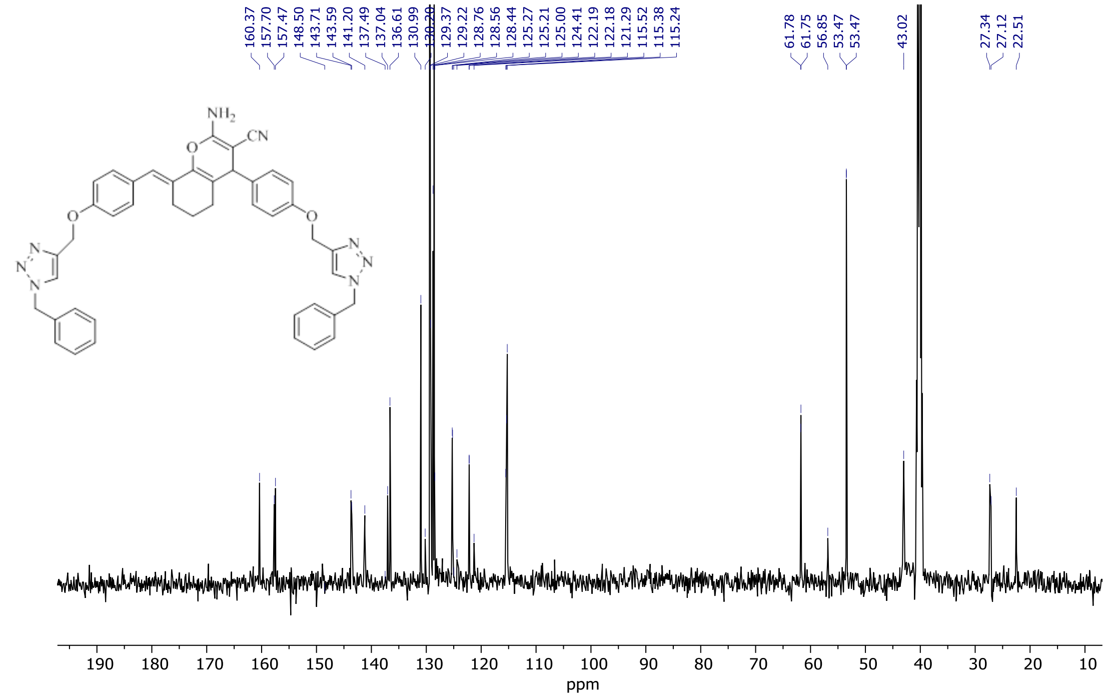

^{13}C NMR (125 MHz, DMSO-d_6) spectrum of compound (*E*)-2-amino-8-(4-((1-benzyl-1*H*-1,2,3-triazol-4-yl)methoxy)benzylidene)-4-(4-((1-benzyl-1*H*-1,2,3-triazol-4-yl)methoxy)phenyl)-5,6,7,8-tetrahydro-4*H*-chromene-3-carbonitrile (**5a**)

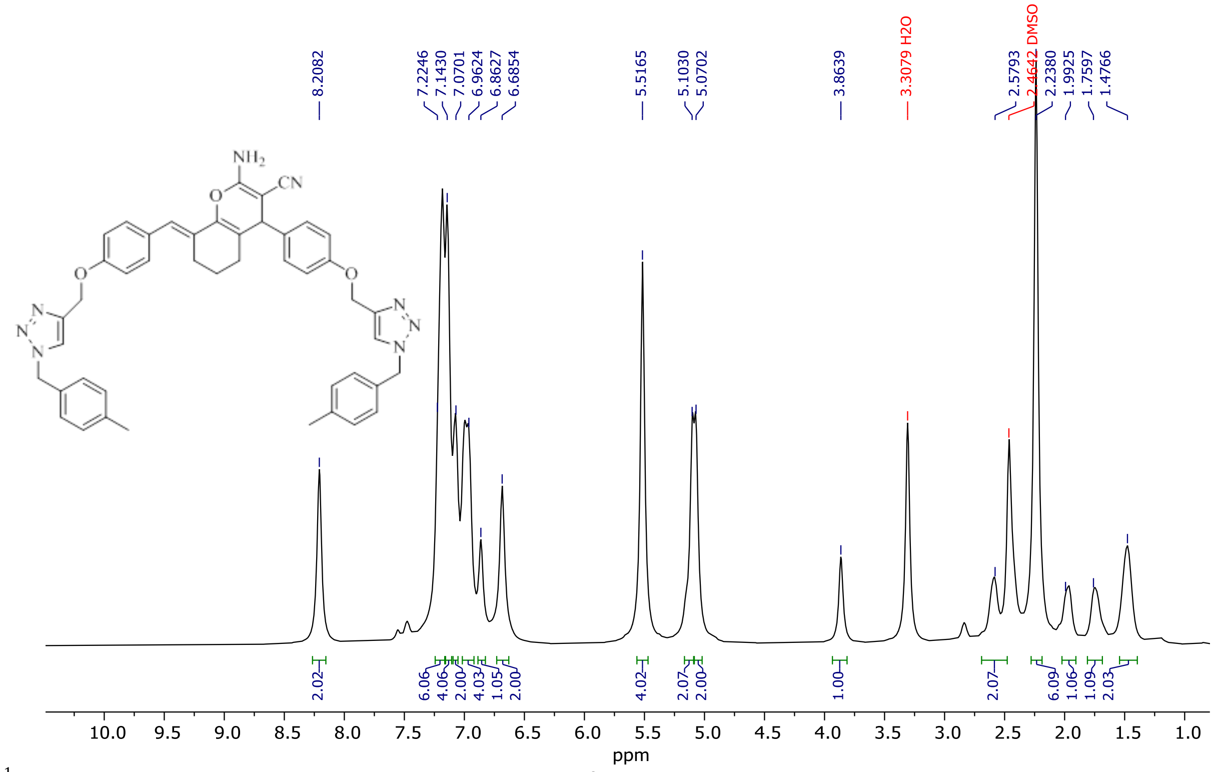

^{1}H NMR (500 MHz, DMSO-d_6) spectrum of (*E*)-2-amino-8-(4-((1-(4-methylbenzyl)-1*H*-1,2,3-triazol-4-yl)methoxy)benzylidene)-4-(4-((1-(4-methylbenzyl)-1*H*-1,2,3-triazol-4-yl)methoxy)phenyl)-5,6,7,8-tetrahydro-4*H*-chromene-3-carbonitrile (**5b**)

hm

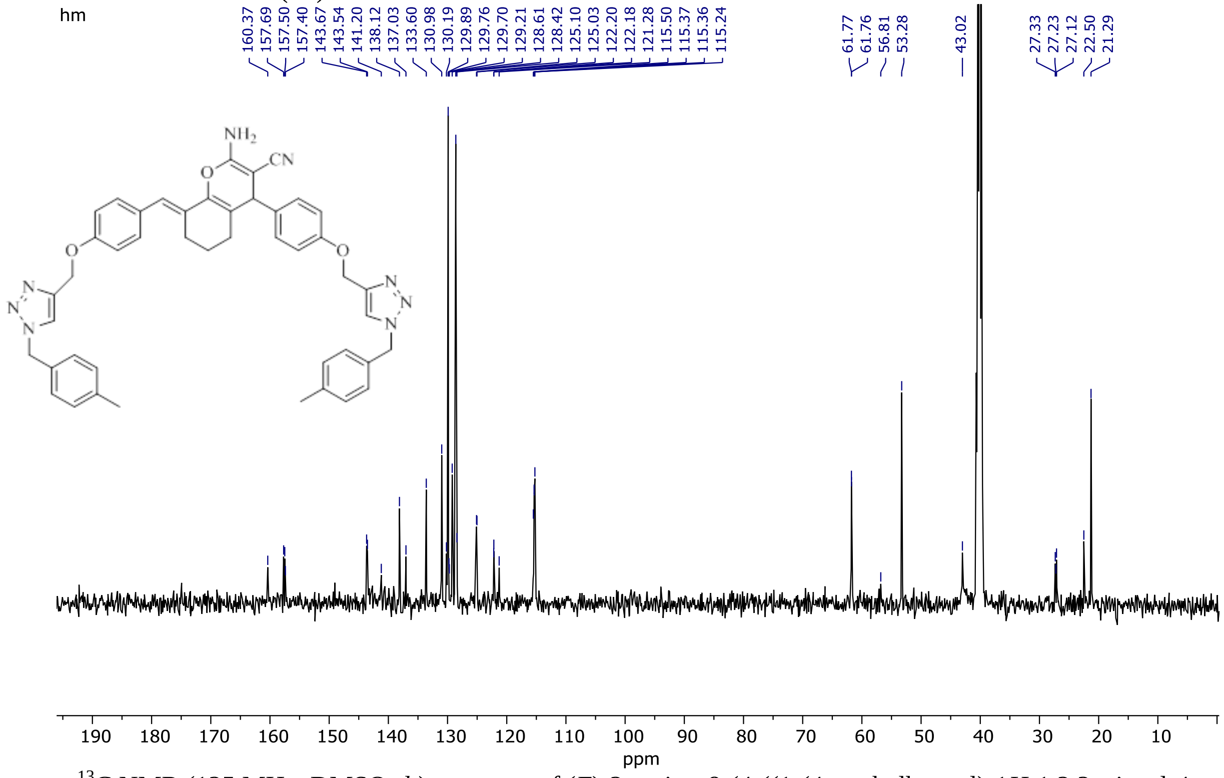

^{13}C NMR (125 MHz, DMSO-d_6) spectrum of (*E*)-2-amino-8-(4-((1-(4-methylbenzyl)-1*H*-1,2,3-triazol-4-yl)methoxy)benzylidene)-4-(4-((1-(4-methylbenzyl)-1*H*-1,2,3-triazol-4-yl)methoxy)phenyl)-5,6,7,8-tetrahydro-4*H*-chromene-3-carbonitrile (**5b**)

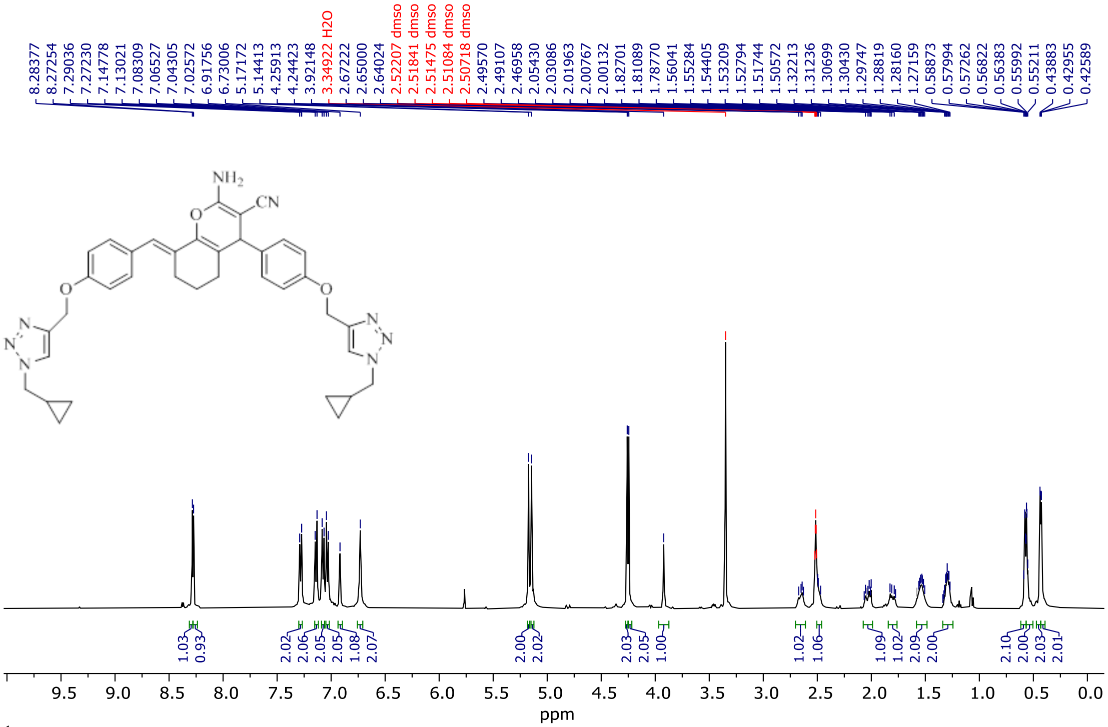

^{1}H NMR (500 MHz, DMSO-d_6) spectrum of (E)-2-amino-8-(4-((1-(cyclopropylmethyl)-1H-1,2,3-triazol-4-yl)methoxy)benzylidene)-4-(4-((1-(cyclopropylmethyl)-1H-1,2,3-triazol-4-yl)methoxy)phenyl)-5,6,7,8-tetrahydro-4H-chromene-3-carbonitrile (**5c**)

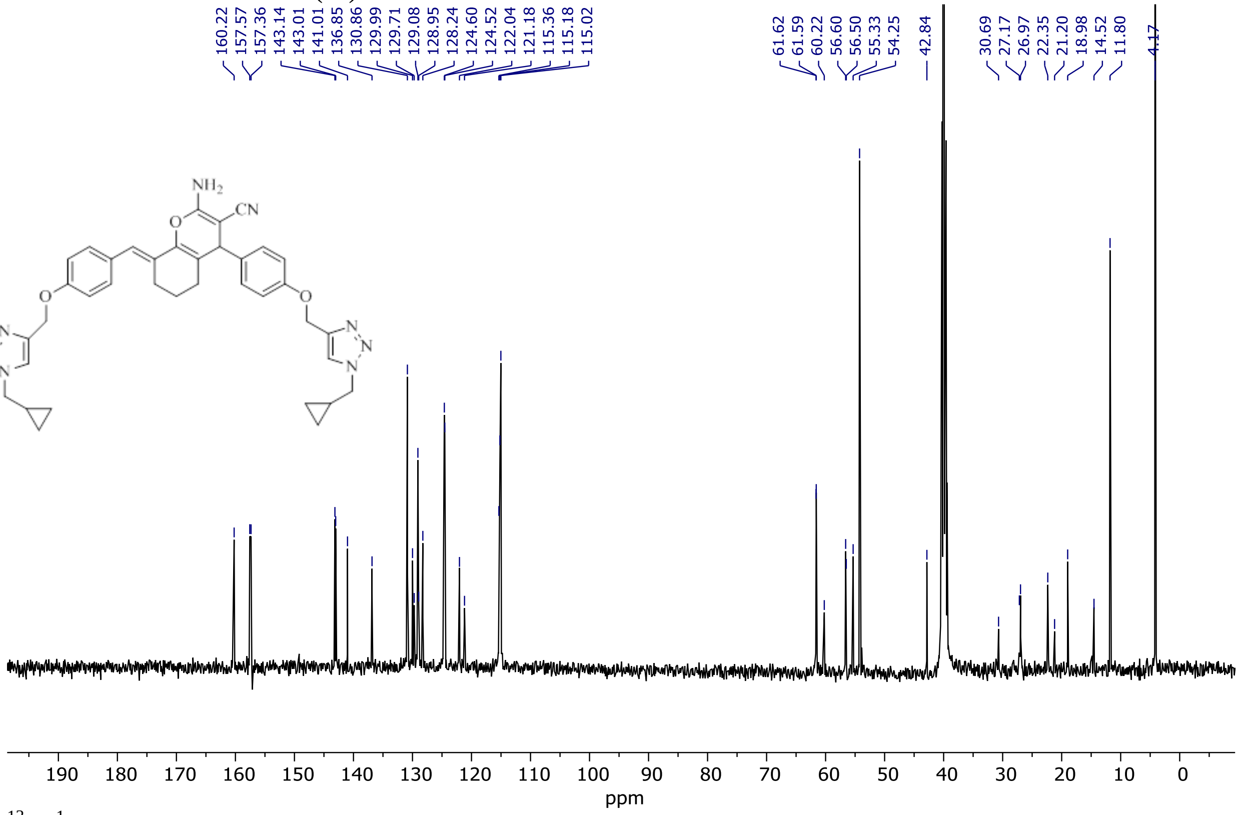

^{13}C ^{1}H NMR (125 MHz, DMSO-d_6) spectrum of (E)-2-amino-8-(4-((1-(cyclopropylmethyl)-1H-1,2,3-triazol-4-yl)methoxy)benzylidene)-4-(4-((1-(cyclopropylmethyl)-1H-1,2,3-triazol-4-yl)methoxy)phenyl)-5,6,7,8-tetrahydro-4H-chromene-3-carbonitrile (**5c**)

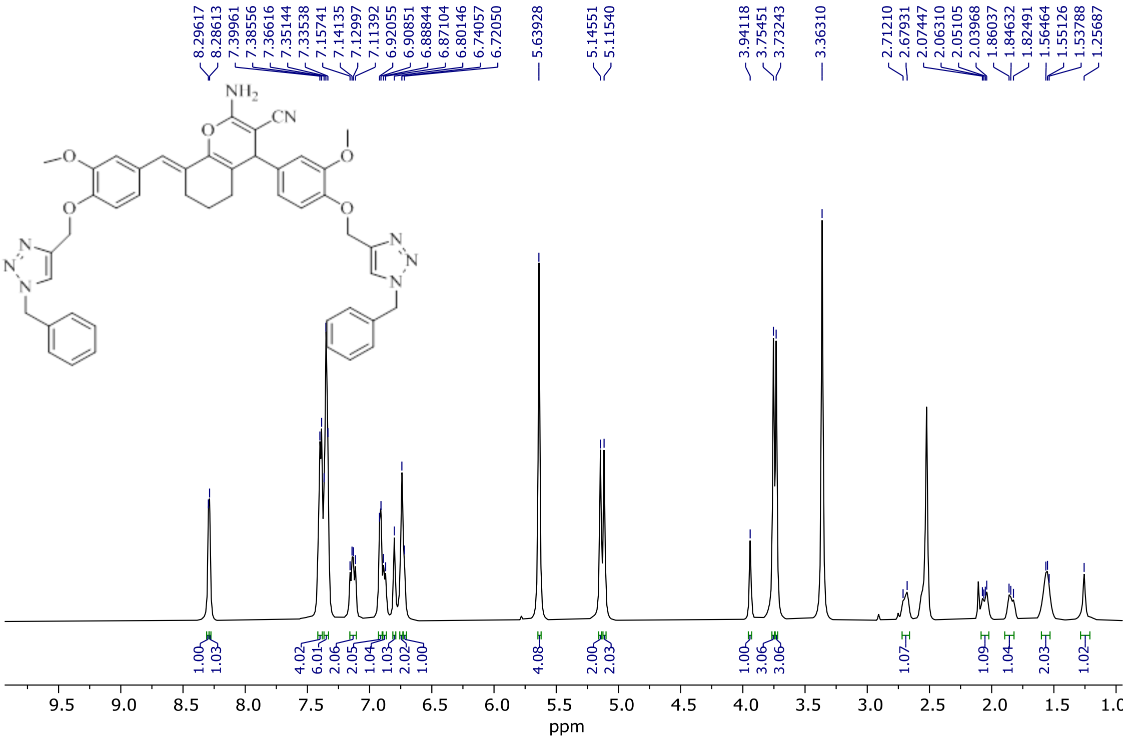

^{1}H NMR (500 MHz, DMSO-d_6) spectrum of (*E*)-2-amino-8-(4-((1-benzyl-1*H*-1,2,3-triazol-4-yl)methoxy)-3-methoxybenzylidene)-4-(4-((1-benzyl-1*H*-1,2,3-triazol-4-yl)methoxy)-3-methoxyphenyl)-5,6,7,8-tetrahydro-4*H*-chromene-3-carbonitrile (**5d**)

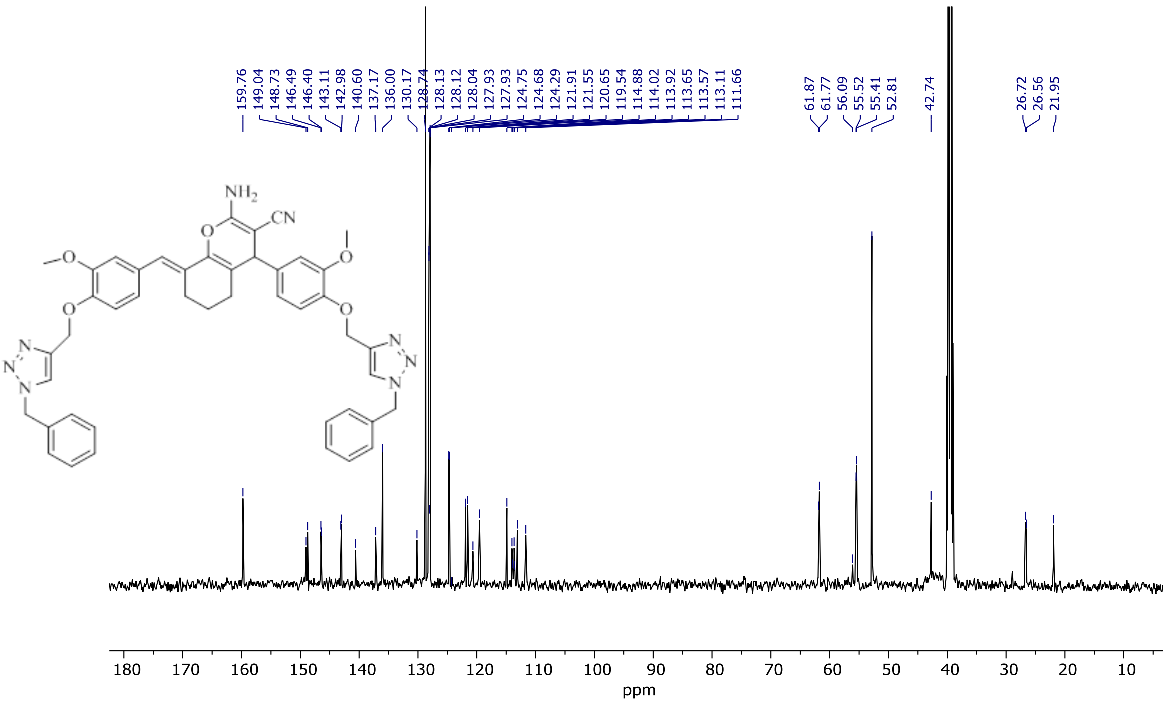

^{13}C NMR (125 MHz, DMSO-d_6) spectrum of (*E*)-2-amino-8-(4-((1-benzyl-1*H*-1,2,3-triazol-4-yl)methoxy)-3-methoxybenzylidene)-4-(4-((1-benzyl-1*H*-1,2,3-triazol-4-yl)methoxy)-3-methoxyphenyl)-5,6,7,8-tetrahydro-4*H*-chromene-3-carbonitrile (**5d**)

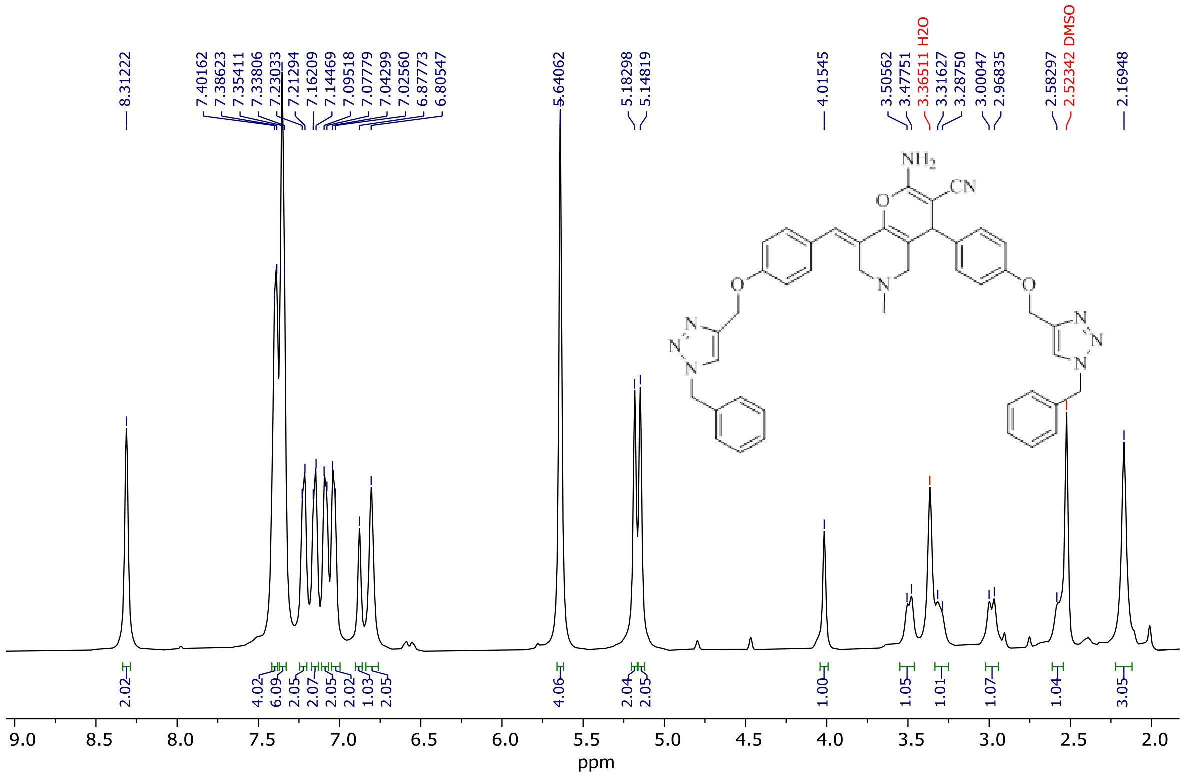

^{1}H NMR (500 MHz, DMSO-d_6) spectrum of (*E*)-2-amino-8-(4-((1-benzyl-1*H*-1,2,3-triazol-4-yl)methoxy)benzylidene)-4-(4-((1-benzyl-1*H*-1,2,3-triazol-4-yl)methoxy)phenyl)-6-methyl-5,6,7,8-tetrahydro-4*H*-pyrano[3,2-c]pyridine-3-carbonitrile (**5e**)

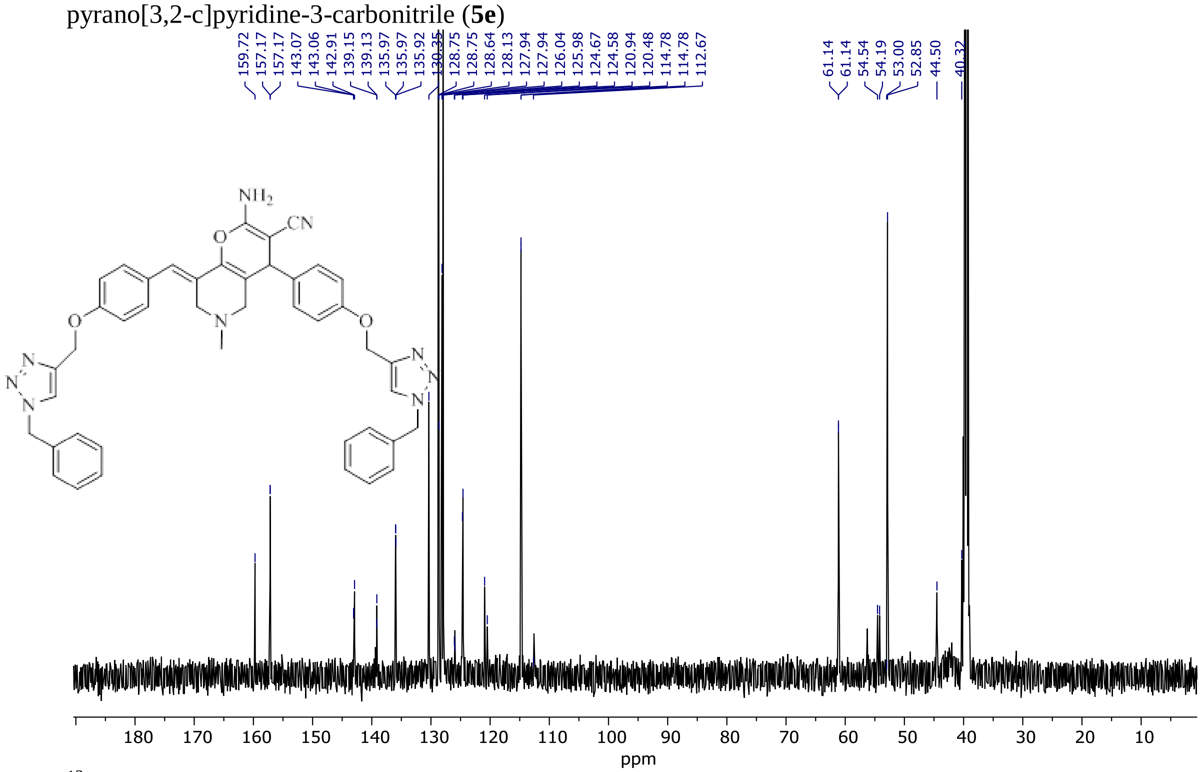

^{13}C NMR (125 MHz, DMSO-d_6) spectrum of (*E*)-2-amino-8-(4-((1-benzyl-1*H*-1,2,3-triazol-4-yl)methoxy)benzylidene)-4-(4-((1-benzyl-1*H*-1,2,3-triazol-4-yl)methoxy)phenyl)-6-methyl-5,6,7,8-tetrahydro-4*H*-pyrano[3,2-c]pyridine-3-carbonitrile (**5e**)

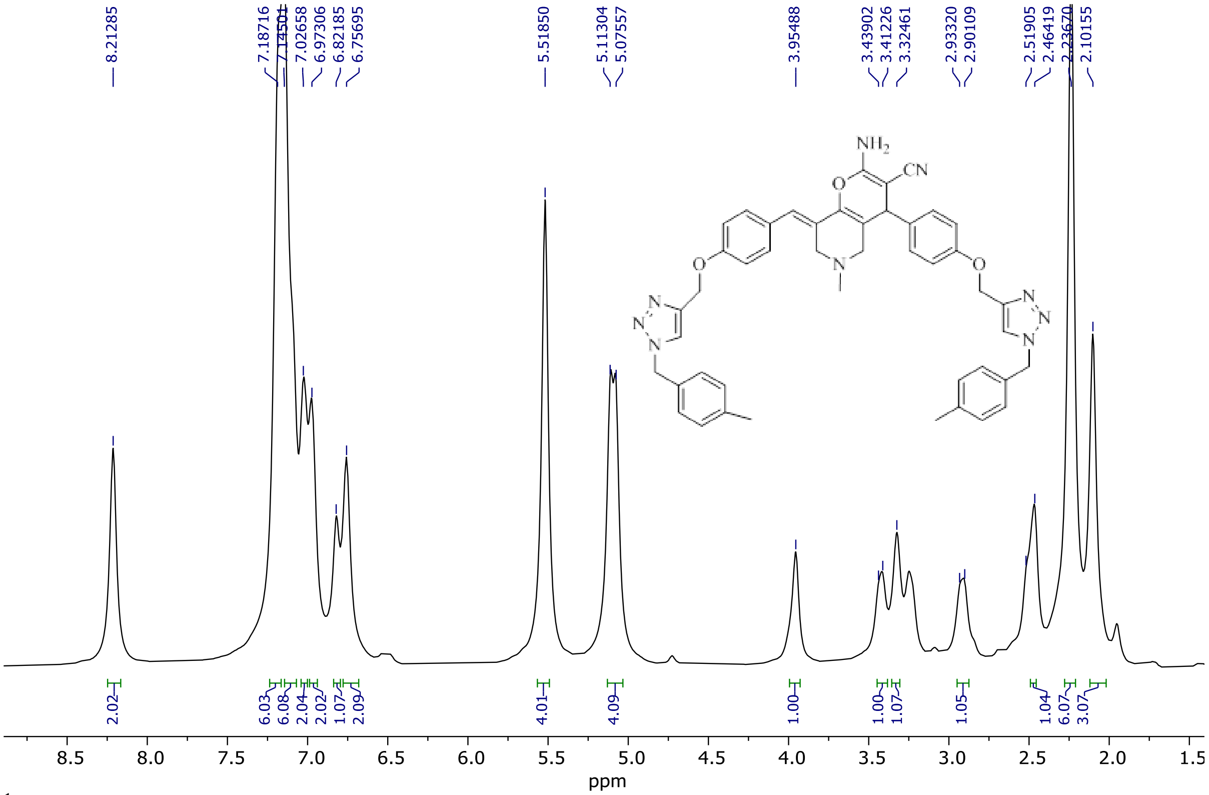

^{1}H NMR (500 MHz, DMSO-d_6) spectrum of (*E*)-2-amino-6-methyl-8-(4-((1-(4-methylbenzyl)-1*H*-1,2,3-triazol-4-yl)methoxy)benzylidene)-4-(4-((1-(4-methylbenzyl)-1*H*-1,2,3-triazol-4-yl)methoxy)phenyl)-5,6,7,8-tetrahydro-4*H*-pyrano[3,2-c]pyridine-3-carbonitrile (**5f**)

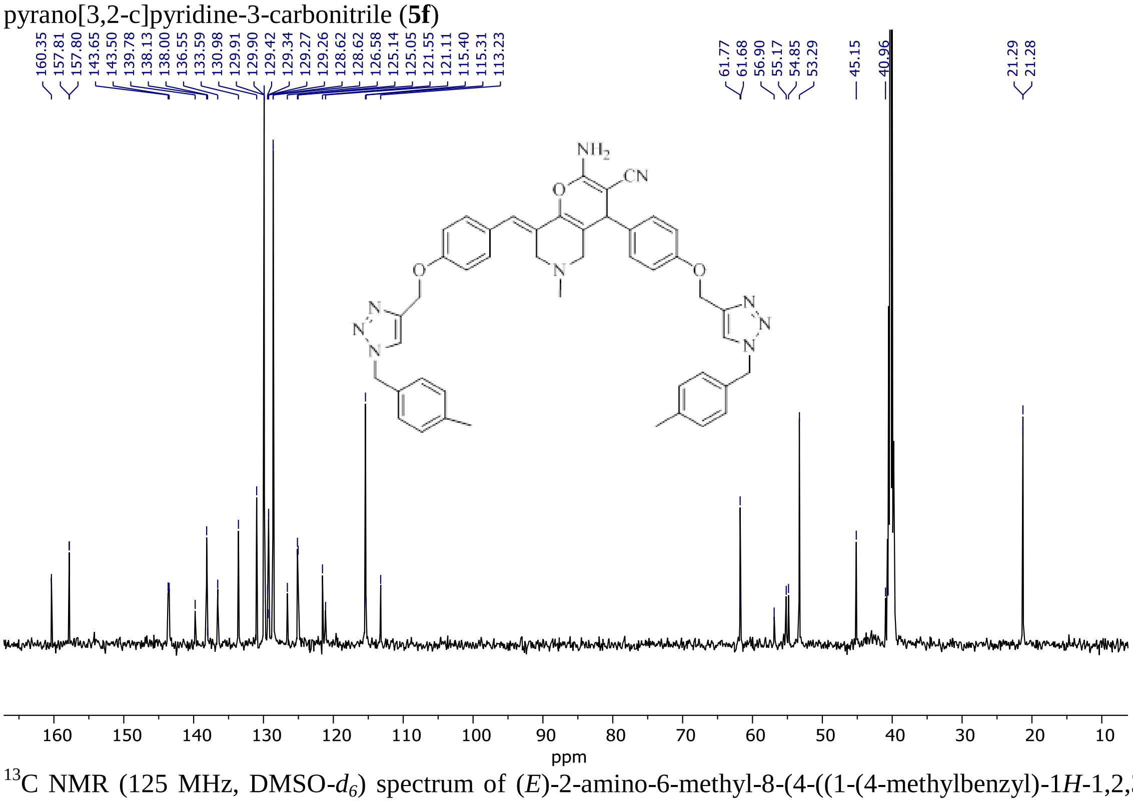

^{13}C NMR (125 MHz, DMSO-d_6) spectrum of (*E*)-2-amino-6-methyl-8-(4-((1-(4-methylbenzyl)-1*H*-1,2,3-triazol-4-yl)methoxy)benzylidene)-4-(4-((1-(4-methylbenzyl)-1*H*-1,2,3-triazol-4-yl)methoxy)phenyl)-5,6,7,8-tetrahydro-4*H*-pyrano[3,2-c]pyridine-3-carbonitrile (**5f**)

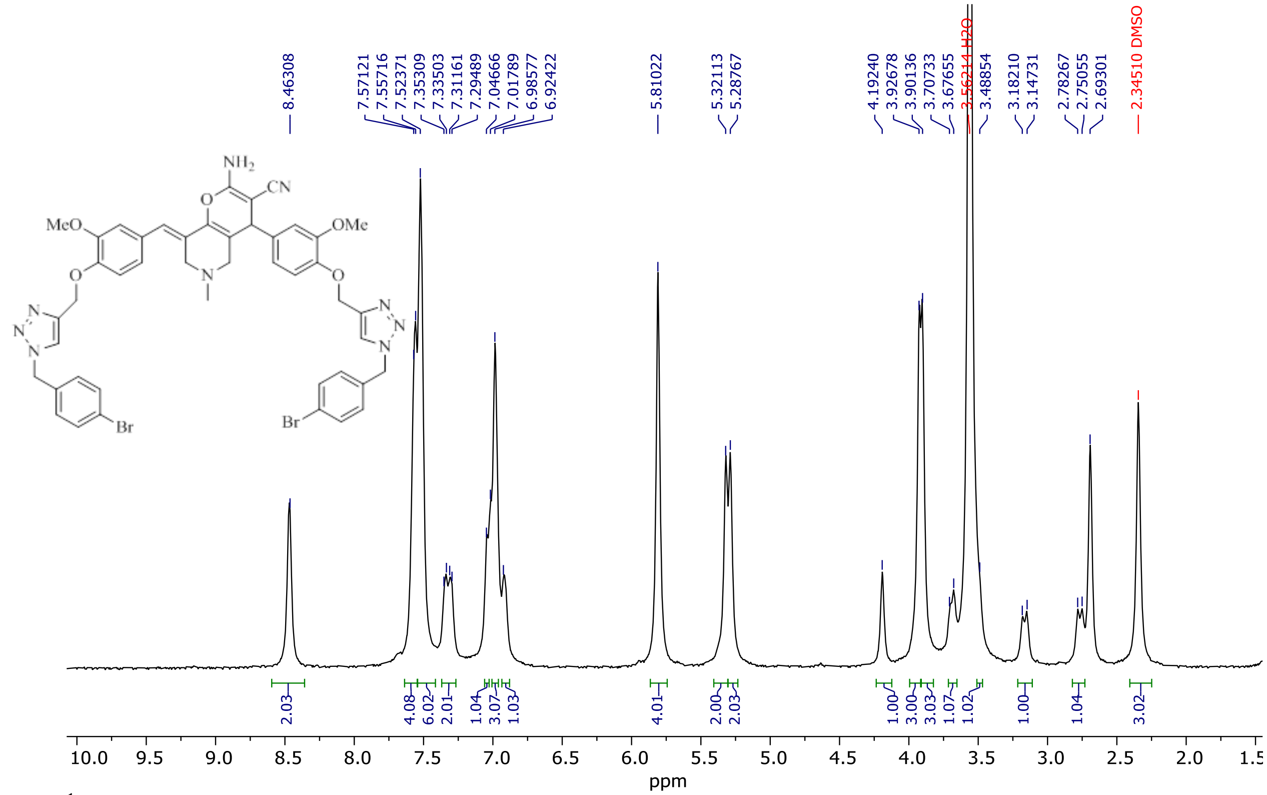

^{1}H NMR (500 MHz, DMSO-d_6) (*E*)-2-amino-8-(4-((1-(4-bromobenzyl)-1*H*-1,2,3-triazol-4-yl)methoxy)-3-methoxybenzylidene)-4-(4-((1-(4-bromobenzyl)-1*H*-1,2,3-triazol-4-yl)methoxy)-3-methoxyphenyl)-6-methyl-5,6,7,8-tetrahydro-4*H*-pyrano[3,2-c]pyridine-3-carbonitrile (**5g**)

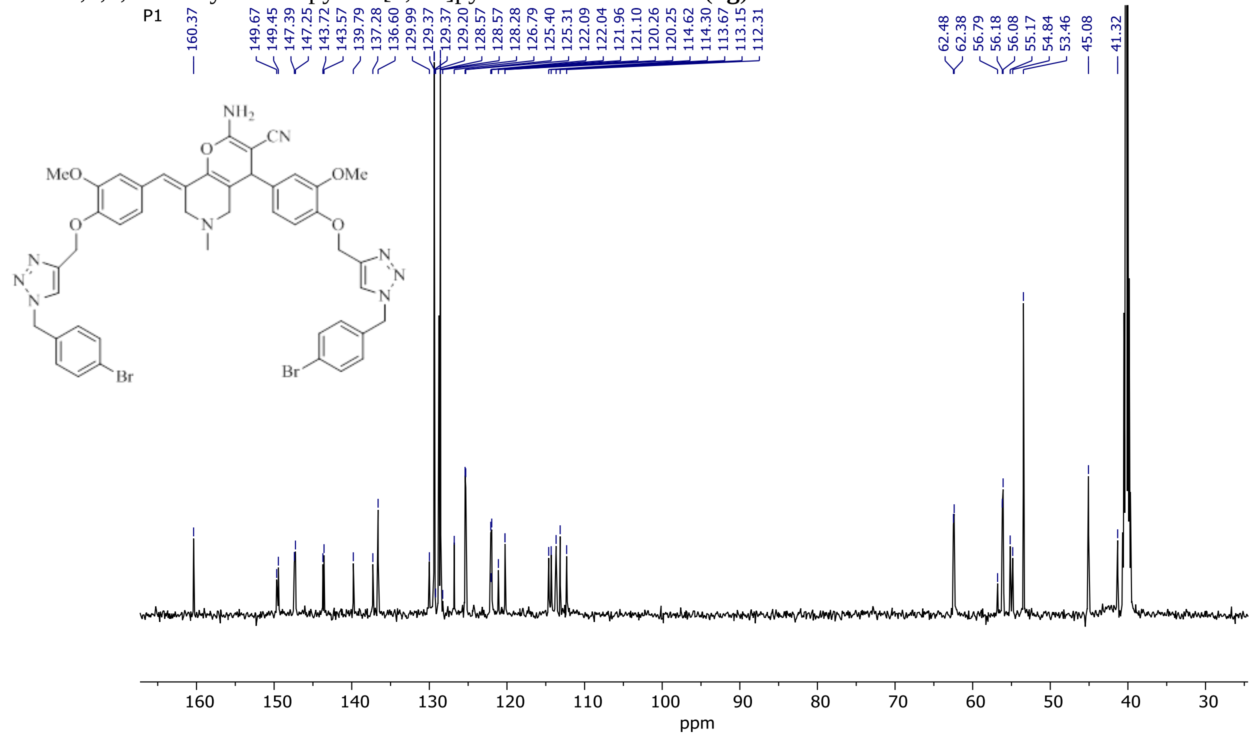

^{13}C ^{1}H NMR (125 MHz, DMSO-d_6) spectrum of (*E*)-2-amino-8-(4-((1-(4-bromobenzyl)-1*H*-1,2,3-triazol-4-yl)methoxy)-3-methoxybenzylidene)-4-(4-((1-(4-bromobenzyl)-1*H*-1,2,3-triazol-4-yl)methoxy)-3-methoxyphenyl)-6-methyl-5,6,7,8-tetrahydro-4*H*-pyrano[3,2-c]pyridine-3-carbonitrile (**5g**)

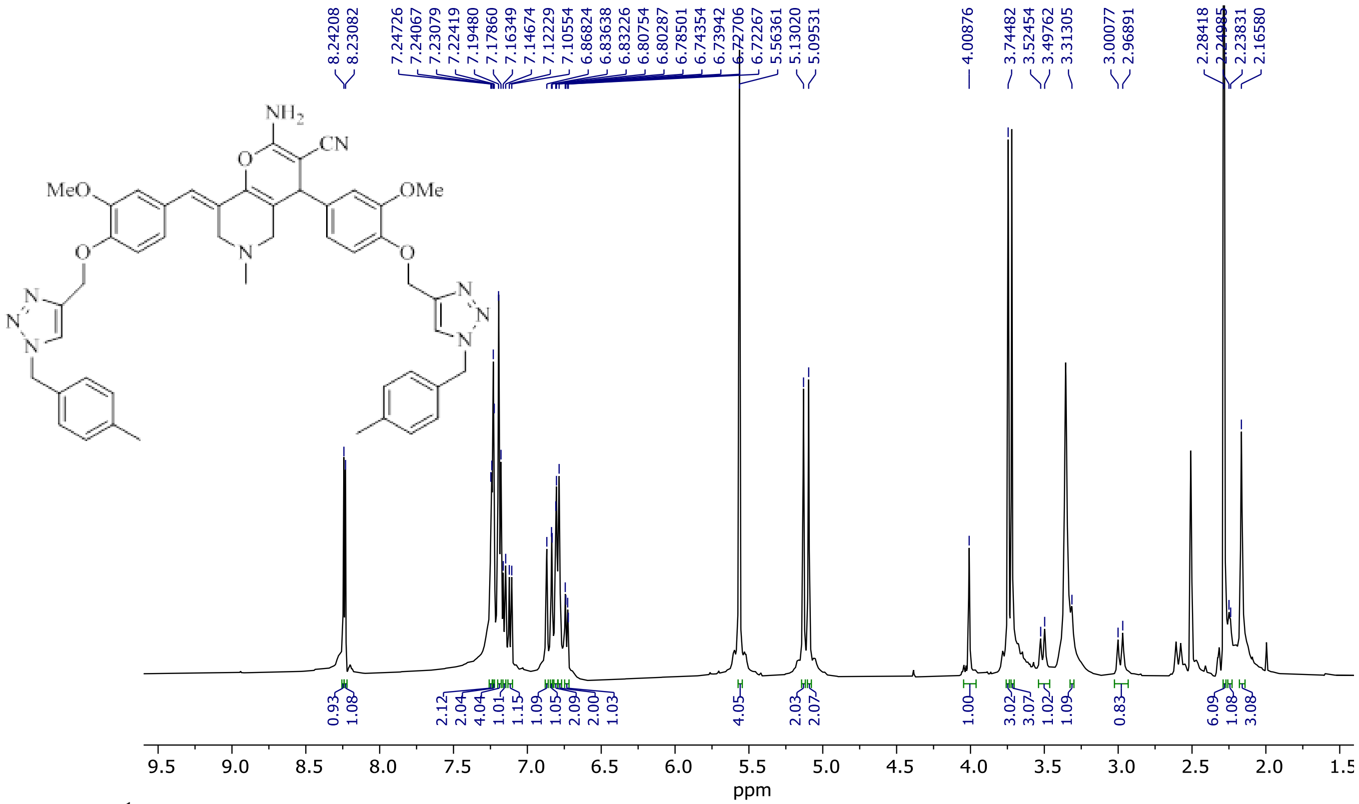

^{1}H NMR (500 MHz, DMSO-d_6) spectrum of (*E*)-2-amino-8-(3-methoxy-4-((1-(4-methylbenzyl)-1*H*-1,2,3-triazol-4-yl)methoxy)benzylidene)-4-(3-methoxy-4-((1-(4-methylbenzyl)-1*H*-1,2,3-triazol-4-yl)methoxy)phenyl)-6-methyl-5,6,7,8-tetrahydro-4*H*-pyrano[3,2-c]pyridine-3-carbonitrile (**5h**)

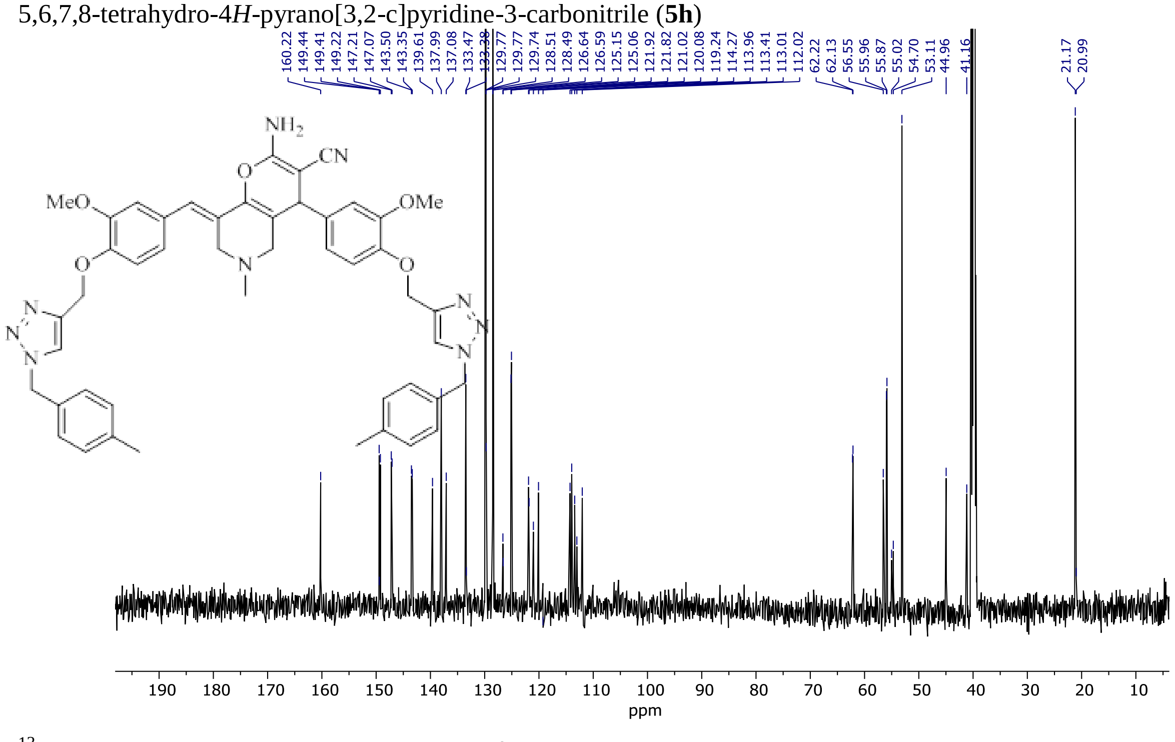

^{13}C NMR (125 MHz, DMSO-d_6) spectrum of (*E*)-2-amino-8-(3-methoxy-4-((1-(4-methylbenzyl)-1*H*-1,2,3-triazol-4-yl)methoxy)benzylidene)-4-(3-methoxy-4-((1-(4-methylbenzyl)-1*H*-1,2,3-triazol-4-yl)methoxy)phenyl)-6-methyl-5,6,7,8-tetrahydro-4*H*-pyrano[3,2-c]pyridine-3-carbonitrile (**5h**)
